# Vesicular Stomatitis Virus Polymerase's Strong Affinity to Its Template Suggests Exotic Transcription Models

**DOI:** 10.1371/journal.pcbi.1004004

**Published:** 2014-12-11

**Authors:** Xiaolin Tang, Mourad Bendjennat, Saveez Saffarian

**Affiliations:** 1 Department of Physics and Astronomy, University of Utah, Salt Lake City, Utah, United States of America; 2 Center for Cell and Genome Science, University of Utah, Salt Lake City, Utah, United States of America; 3 Department of Biology, University of Utah, Salt Lake City, Utah, United States of America; Rutgers University, United States of America

## Abstract

Vesicular stomatitis virus (VSV) is the prototype for negative sense non segmented (NNS) RNA viruses which include potent human and animal pathogens such as Rabies, Ebola and measles. The polymerases of NNS RNA viruses only initiate transcription at or near the 3′ end of their genome template. We measured the dissociation constant of VSV polymerases from their whole genome template to be 20 pM. Given this low dissociation constant, initiation and sustainability of transcription becomes nontrivial. To explore possible mechanisms, we simulated the first hour of transcription using Monte Carlo methods and show that a one-time initial dissociation of all polymerases during entry is not sufficient to sustain transcription. We further show that efficient transcription requires a sliding mechanism for non-transcribing polymerases and can be realized with different polymerase-polymerase interactions and distinct template topologies. In conclusion, we highlight a model in which collisions between transcribing and sliding non-transcribing polymerases result in release of the non-transcribing polymerases allowing for redistribution of polymerases between separate templates during transcription and suggest specific experiments to further test these mechanisms.

## Author Summary

RNA dependent RNA Polymerases tight association with their template creates an almost infinite dilution transcription machinery. Polymerases are delivered to the host cytoplasm associated with the genome template, however, they initiate transcription only at or near the 3′ end of the genome template. How these polymerases initiate and sustain transcription is completely unknown. Given the efficiency of these polymerases and their nontrivial template interactions, understanding their mechanism has both medical and nano-technological applications. Here we show that efficient transcription requires a sliding mechanism for non-transcribing polymerases and can be realized with different polymerase-polymerase interactions.

## Introduction

Transcription is the process of polymerase driven synthesis of mRNA from the genome template. In eukaryotic cells, polymerases engage their promoters through 3D diffusion [1], [2] and have a dissociation constant from their promoters in the range of 40–60 nM [3], [4], [5]. In many viral infections, transcription is the first step for efficient replication. Many viruses, however, do not rely on cellular polymerases for transcription. Specifically non segmented negative strand (NNS) RNA viruses which include potent human pathogens e.g. Rabies, Ebola and measles, deliver special RNA dependent RNA polymerases to transcribe and replicate their genome template [6], [7], [8]. Transcription initiates only at or near the 3′ end of the genome template [9], [10], [11] which immediately poses the question of initiation and sustainability of transcription during early stages of infection.

Here we focus on vesicular stomatitis virus (VSV) which is a prototype of NNS RNA viruses. VSV genomic RNA encodes the 5 viral proteins in the order 3′-N-P-M-G-L-5′ and is fully encapsidated by 1,250 copies of nucleoproteins (N) to form the genome template (N-RNA) [12], [13]. Transcription and following genome replication of VSV is very efficient. Within the first hour of VSV infection, a single template is estimated to yield at least one round of transcription all the way through the L gene with substantially higher transcripts released for the upstream genes [14], [15]. Within 10 hours post infection, a single cell is estimated to produce ∼10,000 progeny VSV virions [16].

The catalytic unit of the VSV polymerase is the L protein, 50 copies of which are packaged within the virion along a single genome template [17]. While the L protein can initiate transcription from its naked genomic RNA [18], it is unable to bind and process the genome template. To transcribe the genome template, L requires association with phosphoprotein P to form a functioning polymerase unit [6], [19] reviewed in [8]. The exact copies of P associated with L during each stage of transcription and replication remains unclear, however in total 400 copies of P are incorporated within each virus [17].

Understanding the position of polymerases along the genome template may provide important clues as to the mechanism of transcription initiation. Polymerases remain associated with the N-RNA templates released from detergent-disrupted virions. When these purified templates are examined with immunogold electron microscopy, the polymerases are found to be almost randomly distributed along the genome template [20]. Within intact virions, Cryo-EM measurements have shown that the 5′ end of the genome template resides at the blunt end of the bullet shaped VSV virion [13] and more recently high resolution fluorescence microscopy also localized polymerases at the blunt end of the VSV virions [21]. Therefore, in combination these measurements localize the polymerases to the 5′ end of the genome template. The mechanism by which polymerases can redistribute along the genome template when extracted from detergent-disrupted virions in the absence of transcription is not well understood.

The polymerases initiate transcription either at the 3′ end of the genome template [10] which results in synthesizing a ∼50 nt leader RNA before synthesizing the N gene or start the synthesis directly at the start of the N gene [9], [11]. Both of these transcription initiation sites (TIS) are located at or near the 3′ end of the genome template and in our manuscript we will not distinguish between initiation at either of these two positions and therefore refer to them collectively as TIS. After initiation, polymerases transcribe the genes coded on the RNA genome starting from the 3′ end in a sequential form [22]. There is a drop of ∼30% in transcription at each gene junction which yields the highest transcription for the N gene and lowest transcription for L [15] (reviewed in [23]). Given that within the first hour of transcription on average at least one L mRNA is synthesized and there is a 30% reduction of transcription at each gene junction [15], a lower bound estimate of the number of N mRNA can be calculated as 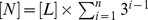 where n is the number of genes on the genome. In case of VSV, n = 5 and therefore the number of N mRNA synthesized at the first hour would be ∼120. Although the attenuation rates are known, the mechanistic details of how polymerases reach the 3′ end and initiate transcription are not clear.

One plausible mechanism to initiate transcription is for a few polymerases to dissociate from the genome template during its delivery to the cytoplasm. The genome template is delivered to the cytoplasm after G protein facilitated fusion of viral envelope with endocytic membranes [24], [25]. During entry, virion interior acidifies, facilitating release of the matrix protein M from the genome template [26]. If some polymerases dissociate during the entry process, they would be able to bind at or near the 3′ end followed by transcription of the genome template [8]. Sustained transcription is dependent on supplying polymerases to the TIS at 3′ end of the genome template, which in principle can happen through dissociation of a fraction of transcribing polymerases at the gene junctions and their subsequent binding to the TIS though 3D diffusion. Although this simple mechanism is attractive, it is difficult to know a priori if such mechanism would be capable to initiate and sustain transcription. The exact topology of the genome is also not clear, genome templates of VSV are assumed to be linear although some circular topologies have been observed through electron microscopy [27]. Since there are only 50 polymerases associated with a single template and rates of transcription of the polymerases are known [15], plausibility of early transcription models with different mechanisms and genome topologies can be tested based on predicted N mRNA levels at the end of one hour of transcription in Monte-Carlo simulations.

Here we constructed Monte Carlo simulations to gauge the fitness of various early transcription models based on their N-mRNA production levels after one hour of transcription. In total 20,000 simulations of early transcription were performed testing a range of polymerase dissociation rates, sliding rates, TIS binding strengths, linear and circular topologies of the genome template and two different polymerase–polymerase collision conditions. Our simulations show that the initial distribution of polymerases is not critical since the transcription machinery rapidly reaches a steady state. We identify the following mechanisms that can sustain transcription and are consistent with our measurements of the dissociation constant of polymerases from their genome template: i) Transcription through sliding facilitated polymerase release, in which polymerases are released from the template through their collision with a transcribing polymerase and engage the TIS through 3D diffusion; ii) Transcription through sliding facilitated initiation on circular templates, in which polymerases find the TIS through 1D sliding on a circular genome template; iii) Transcription through sliding and non-colliding polymerases (in which transcribing and non-transcribing polymerases can pass each other). At the end we propose that sliding of non-transcribing polymerases plays a critical role in VSV transcription. Specifically we highlight the sliding facilitated polymerase release model and discuss how the predictions of this model are consistent with low dissociation constant of polymerases under no transcription and the previously observed redistribution of polymerases between templates during transcription [28]. We conclude that the transcription machinery of NNS RNA viruses is capable of functioning at almost infinite dilutions. We further suggest specific experiments to narrow possible mechanisms.

## Results

We measured the dissociation constant of L from the whole genome template by detergent disrupting the VSV virions and separating the N-RNA template bound with P and L proteins from the rest of the viral proteins using a glycerol cushion spin as detailed in the (S1 Text). The L protein hosts the catalytic site which performs the RNA dependent RNA polymerization and binds the genome template through the phosphoprotein P [8]. Western blot and protein gel analysis was used to detect a small fraction of free L, from which we calculated the dissociation constant to be 20 pM (S1 Text, S1 Figure and S2 Figure). It is important to note that this rate is calculated between the L and the full genome template bound with P and L extracted from detergent-disrupted virions. Since L binds the genome template through its interactions with P, the presence of excess P bound to the N-RNA template produces many L binding sites along the N-RNA templates. We will present an estimate of the dissociation from a single binding site during the discussion. Regardless, the low dissociation constant of the L from the full template guarantees that active polymerases remain bound to the template even when a single template is delivered to the host cytoplasm.

### Linear genome with colliding polymerases

**Figure 1:**
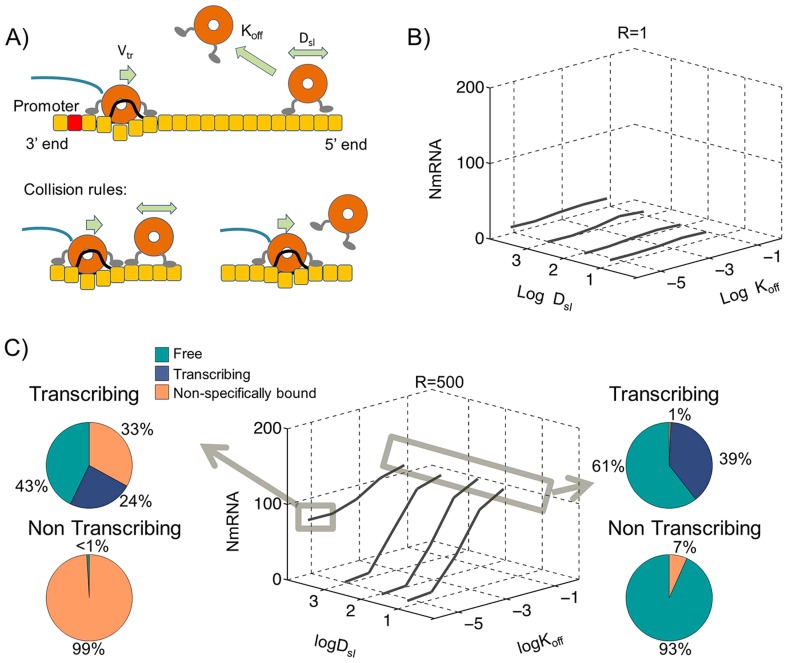
Transcription of linear genome with colliding polymerases. Monte Carlo simulations of 50 polymerases on a linear genome template, (A) shows a representation of this model with collision rules resulting in release of non-transcribing polymerases after collisions. (B) The calculated N mRNA amounts within one hour of simulations for various sliding D_sl_ and dissociation K_off_ rates with relative TIS binding affinity R = 1. (C) Similar simulation results as in B except with R = 500. The highlighted results at left show transcription under sliding facilitated polymerase release (D_sl_ = 10^4^ nm^2^/s and K_off_ = 10^−5^/s) with polymerase distributions showing in pie charts (Top) under transcribing and (Bottom) under non-transcribing conditions. The right panel shows transcription under high polymerase fall off rates with K_off_ = 10^−1^/s.

We simulated transcription from a set of 50 polymerases on the linear genome template using the Monte Carlo rules explained in the methods. In brief, 50 polymerases were followed on the template. Polymerases would only initiate transcription from the TIS at the 3′ end. During transcription, polymerases had a 30% probability of falling off the template at the end of each gene. Non-transcribing polymerases would slide along the template with the 1D diffusion rate D_sl_ and would fall off spontaneously from the genome template with the rate K_off_. In the event of a collision between a transcribing and a non-transcribing polymerase, the non-transcribing polymerase would be forced off the template. Free polymerases in solution would find the template through a diffusion limited reaction and would have a binding affinity R to the TIS relative to a random binding site on the template. Neither K_off_, D_sl_ nor R values are known experimentally, therefore we performed 4,000 simulations spanning a large range of these parameters (D_sl_: 10 nm^2^/s to 10^5^ nm^2^/s; K_off_: 10^−5^/s to 10^−1^/s; R: 1 to 500). We report the measured N mRNA production for each condition as shown in Fig. 1. Initially, polymerases were positioned at the templates 5′ end. The asymmetric initial distribution was compared with random initial distribution which resulted in similar N mRNA productions as shown in S3 Figure. The most critical parameter in this model is the TIS binding strength R; we could not achieve sustained transcription with R = 1 as shown in Fig. 1A. When R was increased to 500, there were two distinct conditions that allowed sustained transcription.

#### Transcription through high dissociation rates of polymerases with no sliding

High fall off rates (K_off_>10^−2^/s) allowed for sustained transcription independent of D_sl_ as shown in Fig. 1C right panel. The insensitivity to sliding is due to interruption with fall off events. This model however has a specific prediction for the fraction of free polymerases in solution both during transcription (61%) and under non transcribing conditions (93%). Specifically, the predicted 93% free polymerases under non transcribing conditions is not supported by our measurements of the low dissociation constant of the polymerases from the template, therefore we do not find this model to be a probable model for sustained transcription.

#### Transcription through sliding facilitated polymerase release

Low fall off rates (K_off_ <10^−3^/s) are incapable of sustaining transcription unless the sliding rate of the non-transcribing polymerases is increased to D_sl_>10^3^ nm^2^/s as shown in Fig. 1. Under the optimum conditions, D_sl_  = 10^4^ nm^2^/s, K_off_ = 6.4×10^−6^/s a total of 72.8±6.2 N mRNA molecules were synthesized. Although in this model polymerases slide readily on the template, examination of polymerase histories showed that only 2% of polymerases found the TIS through sliding, whereas 98% found the TIS through 3D diffusion from solution. From the polymerases that found the TIS through 3D diffusion, 99.7% were dissociated from the template through a collision with a transcribing polymerase. In sliding facilitated polymerase release as highlighted in Fig. 1C left, 99% of polymerases are bound to the template in the absence of transcription. As soon as transcription is turned on, 43% of polymerases get knocked off the template through collisions with transcribing polymerases. These free polymerases then engage the TIS through 3D diffusion to sustain transcription.

### Circular genome with colliding polymerases

The one possible caveat for the single track linear genome model presented in Fig. 1 is the relatively high TIS binding strength (R = 500) required for sustaining transcription. The TIS binding strength in the NNS RNA viruses has not been measured independently. Given the unique structure of the 3′ end of the genome template, it is possible that a high TIS binding strength can exist, but it can also be argued that the direct TIS binding strength may not be so high since the promoter is buried under a layer of N proteins. To create a model that does not require high TIS strength, we made an assumption that the N-RNA template can be circularized. Such circular genome templates have been isolated from VSV infected Hela cells [27], however the biological implications and the mechanism of circularization have remained unclear. By assuming that polymerases which reached the 5′ end could move forward and start at the 3′ end of the template we practically inserted the circular genome template into the MonteCarlo simulations as described in previous sections and methods. Similar to the model presented for the linear genome, any polymerase reaching the TIS could initiate transcription.

#### Transcription through sliding facilitated initiation on circular templates

**Figure 2:**
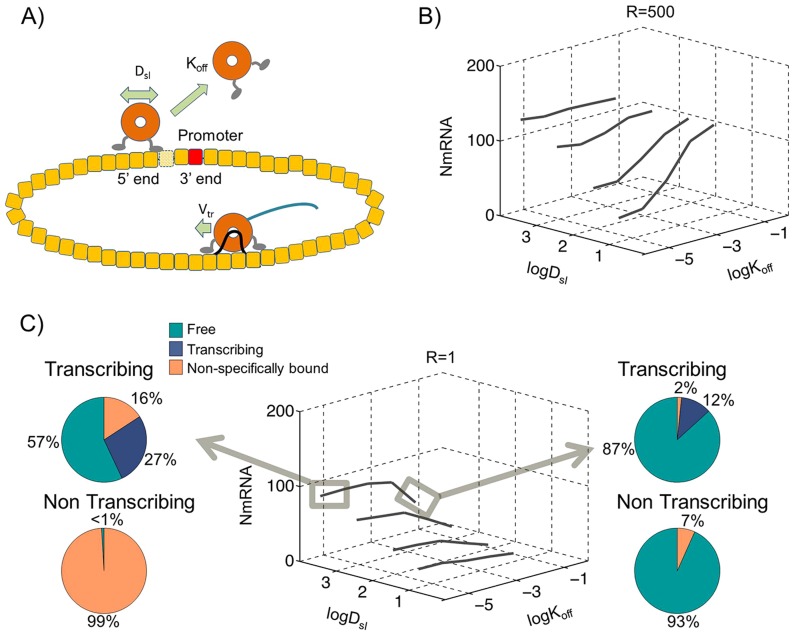
Transcription of circular genome with colliding polymerases. Monte Carlo simulations of 50 polymerases on a circular genome template, (A) shows a representation of this model with collision rules resulting in release of non-transcribing polymerases after collisions. (B) The calculated N mRNA amounts within one hour of simulations for various sliding D_sl_ and dissociation K_off_ rates with relative binding affinity to TIS R = 500 (C) and similar simulation results as in B except with R = 1. The highlighted results at left show transcription through sliding facilitated initiation on circular templates (D_sl_ = 10^4^ nm^2^/s and K_off_ = 10^−5^/s) with polymerase distributions showing in pie charts (Top) under transcribing and (Bottom) under non-transcribing conditions. The right panel shows transcription under high Polymerase fall off rates with K_off_ = 10^−1^/s.

We tested the model with circular templates that included sliding of non-transcribing polymerases with a diffusion coefficient D_sl_ and a fall off rate of K_off_ as shown in Fig. 2. In this model we could recover the transcription even under low TIS binding strength (R≤10) while keeping the polymerases bound to the template when transcription stopped. Under the most favorable conditions R = 1, D_sl_ = 10^4^ nm^2^/s K_off_ = 6.4×10^−6^/s as highlighted in Fig. 2C left panel, a total of 80.6±6.5 N mRNA molecules were synthesized in one hour of transcription. More careful investigation into the histories of the polymerases in this model revealed that contrary to the sliding facilitated polymerase release model highlighted in Fig. 1C left panel, 99.5% of polymerases found and engaged the TIS through 1D diffusion. Although free polymerases contributed only 0.5% to transcription initiation under the circular genome model, 99% of the free polymerases were still released into the solution through collision with transcribing polymerases. The presence of free polymerases during transcription combined by their tight association under non-transcribing conditions is in agreement with results showing redistribution of polymerases from UV irradiated templates [28].

### Linear genome with non-colliding polymerases

The collision between transcribing and non-transcribing polymerases is not trivial. It is possible that the transcribing polymerases may be capable of bypassing a non-transcribing polymerase without dissociating the non-transcribing polymerase from the template. Although the RNA is buried under N protein and cannot form secondary structures, it is possible that the N-RNA genome template can form transient kissing loops which would facilitate polymerase transfer from one section of the N-RNA to the next effectively bypassing a transcribing polymerase.

**Figure 3:**
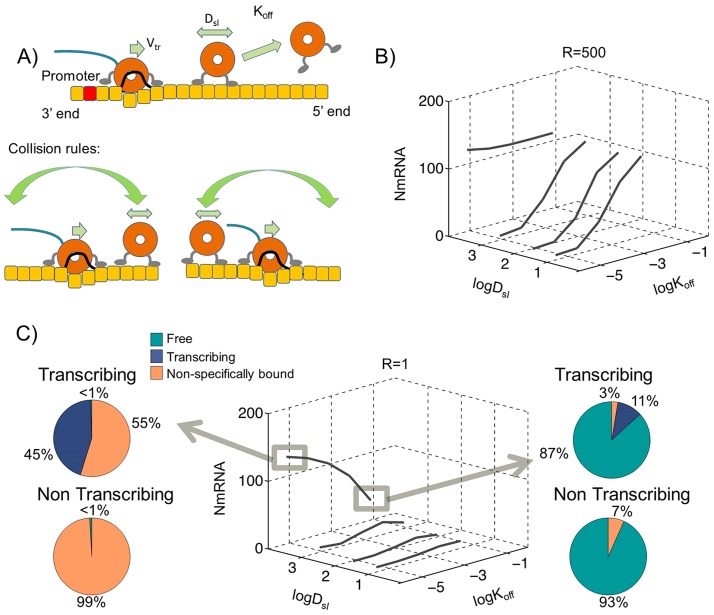
Transcription of linear genome with non-colliding polymerases. Monte Carlo simulations of 50 polymerases on a linear genome template, (A) shows a representation of this model with collision rules resulting in no collisions between non-transcribing and transcribing polymerases. (B) The calculated N mRNA amounts within one hour of simulations for various sliding D_sl_ and dissociation K_off_ rates with relative binding strength to TIS R = 500 and (C) similar simulation results as in (B) except with R = 1. The highlighted results at left show transcription through sliding non-colliding polymerases (D_sl_ = 10^4^ nm^2^/s and K_off_ = 10^−5^/s) with polymerase distributions showing in pie charts (Top) under transcribing and (Bottom) under non-transcribing conditions. The right panel shows transcription under high polymerase fall off rates with K_off_ = 10^−1^/s.

To test these conditions, we changed the collision rules so polymerases can bypass each other and simulated this model by exploring the sliding, dissociation and TIS binding strengths (D_sl_: 10 nm^2^/s to 10^5^ nm^2^/s; K_off_: 10^−5^/s to 10^−1^/s; R: 1 to 500) as shown in Fig. 3. Contrary to the single track model proposed in Fig. 1, the double track model was capable of sustaining transcription both under high R = 500 as well as low R = 1 relative TIS binding strengths as shown in Fig. 3.

As expected under high dissociation rates (K_off_>10^−2^/s) we could observe sustained transcription independent of the sliding diffusion coefficient D_sl_ similar to results shown in Fig. 1 for colliding polymerases.

#### Transcription through sliding and non-colliding polymerases

The main advantage of the non-colliding polymerase model presented in Fig. 3 is its sustained transcription under low TIS strengths given a high enough sliding coefficient. In the optimum conditions as shown in Fig. 3C left panel with D_sl_ = 10^4^ nm^2^/s, K_off_ = 6.4×10^−6^ and R = 1, there were 129±9 N mRNA molecules synthesized within the first hour of transcription. Analysis of the individual polymerase histories after the simulation revealed that 100% of the polymerases initiating transcription arrived at the TIS via 1D sliding. In the non-colliding Polymerase model as presented here, there is a significant absence of free polymerases during transcription as shown in Fig. 3C left panels. This is in contrast to the 43% free polymerases present during transcription in a colliding polymerase model as presented in Fig. 1C left panel.

### A one-time dissociation of polymerases during entry is incapable of sustaining transcription

**Figure 4:**
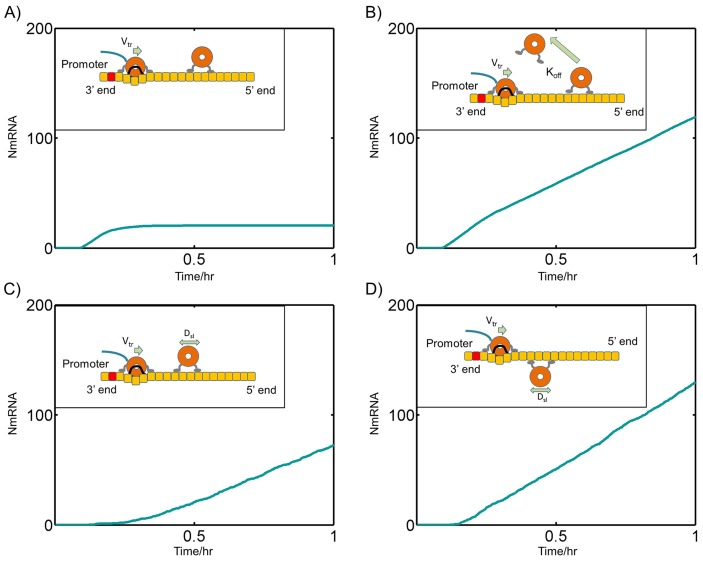
Trajectory of transcription from various models. Plots of N mRNA versus time with linear genome: (A) Initially all polymerases are in solution with D_sl_ = 0, R = 500, K_off_ = 10^−5^/s (B) same as (A) except K_off_ = 10^−1^/s. (C) Transcription through sliding facilitated Polymerase release with R = 500, D_sl_ = 10^4^ nm^2^/s, K_off_ = 10^−5^/s. (D) Transcription through sliding non-colliding polymerases with R = 1, D_sl_ = 10^4^ nm^2^/s, K_off_ = 10^−5^/s.

It can be speculated that transcription can be sustained by a one-time dissociation of polymerases from the template during cellular entry in the absence of sliding. Our simulations however show that such a model is incapable of sustained transcription and polymerases eventually stop transcribing as shown in Fig. 4A. This Fig. also includes the time trajectory of N mRNA production for various other models including transcription through high dissociation rates of polymerases with no sliding as shown in Fig. 4B, Sliding facilitated polymerase release as shown in Fig. 4C and transcription through sliding and non-colliding polymerases as shown in Fig. 4D, all of which except 4A can sustain transcription.

To address the issues of initial conditions further, we have measured the N mRNA production with initial conditions: i) randomly distributed polymerases and ii) asymmetrically distributed polymerases at the 5′ end. These measurements were performed on the same range of K_off_ (from 10^−5^/s to 10^−1^/s) and D_sl_ (from 10 nm^2^/s to 10^5^ nm^2^/s) as shown in S3 Figure produced minimal effects on the overall transcription rates.

## Discussion

The Monte Carlo simulations presented here were used to quantify the transcription efficiency and sustainability of various models. Simple mechanisms like jump starting the transcription with initial dissociation of polymerases during entry did not produce sustainable transcription. The most critical issue for sustaining transcription is to have efficient mechanisms for delivery of polymerases to TIS at the 3′ end. This can be achieved either through 3D diffusion or 1D sliding on the genome template. Below we will discuss specific models presented from our results that can sustain transcription.

### Transcription through high dissociation rates of polymerases

The most simple model that can deliver the polymerases to the 3′ initiation site through 3D diffusion is based on dissociation of non-transcribing polymerases from the genome template. In order for this model to sustain transcription, two conditions need to be satisfied. Like any other model that relies on 3D diffusion, this model requires a high relative affinity for the TIS (R>500) and a high dissociation rate of (K_off_>10^−2^/s). The interesting aspect of this model is that it is insensitive to sliding rates, genome topology and/or collision conditions as shown in Fig. 1C, 2C and 3C right panels. While the TIS binding affinity has not been experimentally measured, the more substantial problem with this model is the large fraction of free polymerases predicted in the absence of transcription in all conditions (Fig. 1C, 2C and 3C right panels). This is inconsistent with our measurements of dissociation constant of polymerases from the genome template as presented in S1 Text and also inconsistent with live imaging visualization of the fluorescently modified P proteins during the replication cycle of VSV in Hela cells, in which most of the P is localized with puncta assumed to be genome templates ([29] and our unpublished observations). The existence of a mechanism for high dissociation rates in vivo cannot be completely dismissed. The live imaging data cited above do not show conclusively that the observed P molecules are in association with genome templates and not less substantial aggregates of N proteins. There also exist substantial differences between transcription initiation in vivo and in vitro [9], [11] and therefore there is a finite possibility that the dissociation constant of the polymerases can be sufficiently increased within the cytoplasm to make this model work. However there is no current data indicating such a possibility and further experiments are required to measure the concentration of free polymerases in the cytoplasm. Therefore we will not highlight this model as the most likely model for transcription.

### Transcription through sliding facilitated polymerase release

When collision conditions between transcribing polymerases and non-transcribing polymerases on a linear template are such that the non-transcribing polymerases get ejected from the template due to this collision, these ejected polymerases can reach the TIS at the 3′ end of the genome given high relative TIS binding affinity (R = 500). This creates an interesting model in which immediately upon halting transcription, all the polymerases would bind back to the genome template as shown in Fig. 1C left panel. In this model, during transcription 43% of polymerases are free in solution and this percentage drops to <1% immediately upon halting transcription. This model is both consistent with our measurements of polymerase dissociation constants under non-transcribing conditions as well as the observed re-distribution of polymerases from UV irradiated templates [28]. In the experiments with UV irradiated templates, genome templates along with associated polymerases were purified from UV irradiated VSV virions. These damaged templates showed a significant reduction of transcription in vitro. In subsequent experiments, genome templates minus their polymerases purified from WT undamaged VSV virions were added to the UV irradiate genome templates bound with polymerases. This mixing resulted in a boost of total transcription indicative of transfer of polymerases from the damaged template to the undamaged template. This experiment is indeed in agreement with the sliding facilitated polymerase release model since this model predicts a significant pool of polymerases are released from the templates due to collision of transcribing and non-transcribing polymerases. The important point being that although the genome templates are UV irradiated, their polymerases are still capable of initiating transcription. This initiation leads to either premature dissociation or creation of a roadblock that facilitates dissociation of sliding non-transcribing polymerases due to collisions. To demonstrate this effect we simulated the transcription from the UV damaged templates as shown in S1 Text S4 Figure and demonstrate that polymerases would fall off the UV irradiated templates. Based on these experiments we highlight the sliding facilitated transcription model as a most likely model of transcription.

### Transcription through sliding facilitated initiation on circular templates

This is the first model we tested in which polymerases would find the initiation site through 1D sliding and are capable of sustaining transcription. The advantage of this model is that it is operational even under low relative binding strength of the TIS (R = 1) as shown in Fig. 2C left panel. Although in this model, similar to the sliding facilitated polymerase release model, >50% of polymerases are free in solution during transcription, careful analysis of the polymerase trajectories in the simulations shows that 99.5% of the polymerases that initiate transcription, find the initiation site through 1D sliding. This model is also consistent with the observed low dissociation rate of the polymerases since in non-transcribing conditions 99% of polymerases remain bound to the templates as shown in Fig. 2C right panel. The major problem with this model is that aside from early electron microscopy data [27], the presence of circular templates during transcription has not been verified experimentally, however this model is consistent with the observed redistribution of polymerases from UV irradiated templates [28] and therefore would be the most likely model of transcription if measurements show a low TIS binding strength.

### Transcription through sliding non-colliding polymerases

If we assume that transcribing polymerases can bypass non-transcribing polymerases on the template and that non-transcribing polymerases can slide on the genome template we arrive at a model that does not require circular templates, sustains a high rate of transcription and can operate with almost no free polymerases in solution either during or under non-transcribing conditions as shown in Fig. 3C left panels. This is an attractive mechanism since it will create an infinite dilution, transcription machinery with no free polymerases even under transcribing conditions. However, the mechanics of polymerase collisions are nontrivial and it is not clear how a non-transcribing polymerase can retain its affiliation to the template under heavy reorganization of the template during passage of a transcribing polymerase [8], [19]. More substantially, since this model predicts almost no dissociation of polymerases during transcription, it fails to explain the previously observed redistribution of polymerases between UV irradiated and undamaged genome templates [28].

### Possible other mechanisms

All the mechanisms represented here and tested in the MonteCarlo simulations are inspired by previously observed mechanisms in DNA enzymes and molecular motors. It is however possible that redistribution of polymerases in NNS RNA viruses has a unique and different mechanism. One such mechanism, can be transferring of the polymerases from one section of the template to the other through collisions between template sections, such a model can be termed effectively the “Spider-Man” model. In such a model, sliding of polymerases would not be essential since polymerases can redistribute along the template through strand transfer collisions. Theoretical prediction of the spider-man model is complicated, since it depends on the three dimensional dynamics of the genome template, which is currently unknown. The spider-man model however is predicted to be heavily dependent on template self collisions and therefore it predicts a severe decrease in polymerase redistributions under stretched template geometries which can be achieved by mechanical manipulations of the template.

### Proposing future experiments

It becomes clear when comparing the panels in Figs. 1, 2 and 3C that one of the main indicators of the various models are the amount of free polymerases during active transcription. Therefore, the essential next step should be to directly measure the amount of free polymerases during transcription. Since transcription can be reconstituted from purified VSV genome templates, these experiments are highly feasible. With the advent of new quantitative fluorescent measurements like Fluorescence Correlation Spectroscopy [30], [31], [32], the minimal amount of free polymerases can also be measured during transcription in live cells, given the proper tagging of fluorescent polymerase components.

Failure to detect free polymerases during transcription would make the sliding non-colliding polymerase mechanism proposed in Fig. 3 likely. Our simulations indicate that sliding of non-transcribing polymerases plays a critical role in all proposed mechanisms. Measurements of this sliding rate in vitro using available single molecule techniques, although possible, is very challenging due to lack of tools for immobilizing the genome template. These measurements however, would be crucial to validate the proposed sliding mechanism in NNS RNA virus transcriptions.

### Implications and comparisons

Given the 20 pM dissociation constant measured for L with its full genome template, how can a sliding model be justified? Indeed, the dissociation constant of molecular motors from their tracks is in the low nanomolar range and the dissociation constant of the T7 RNA polymerase to its class III TIS is in the range of 40–60 nM [3], [4], [5]. Based on our approximate division of the template to 10 nm regions (the size of the polymerase), we conclude that around 300 polymerases could fit onto a single N RNA template. If we assume that all these interactions are linear, we can estimate that a single binding site would have a dissociation constant of 6 nM, which would be much closer to the observed dissociation constants from other procesive motors. The sliding movement of the polymerase is likely realized through transient binding and dissociation of the P protein to the N-RNA, structural evidence of such transient interactions have been previously demonstrated in paramyxoviruses [33].

Cellular DNA based RNA polymerases have been shown to bind directly to their promoters to initiate transcription [1], [2]. Although we incorporate sliding of polymerases along the N-RNA template, our model of transcription in the linear genome also relies on binding of the polymerases through 3D diffusion to a TIS ((R>500) Fig. 1). It is only under the low TIS strength and circular templates that our model predicts polymerases will find the TIS through sliding (Fig. 2). Combinations of sliding and 3D diffusion has been proposed previously for lac repressor facilitated diffusion to reach its target sequences up to 100 fold faster than the diffusion limit [34], [35]. DNA repair enzymes were also shown to utilize long range 1D sliding on template [36], [37]. Sliding along the genome template therefore can be a plausible mechanism.

In NNS RNA viruses, aside from initiation at the 3′ end [10], transcription can be efficiently initiated at the start of the N gene as shown in vivo [9] and in vitro [11]. These observations can be reconciled if one assumes that polymerases can scan the template, therefore always entering at the 3′ end but sometimes scanning their way to the start of the N gene without synthesizing the leader RNA [38], [39]. Scanning is not the only proposed mechanism, a direct binding model based on structural rearrangement of the N proteins around the TIS can also explain the start of initiation directly at the N gene junction [40]. The proposed scanning as discussed above is likely different from the sliding mechanism we propose here since the scanning takes place with a polymerase already engaged with the RNA, while our proposed sliding can happen on the surface of the N-RNA without the need to rearrange the template. The asymmetric localization of polymerases at the 5′ end of the genome within intact virions [41] in conjunction with their random distribution along the genome template when extracted from detergent solubilized virions [20] supports a sliding mechanism by which polymerases can redistribute on the genome template after release from the virion.

In our model we investigated only the first one hour of transcription. The kinetics of transcription during the replication cycle beyond the first hour have been previously modeled for viral infection cycles including: kinetic modeling of viral growth in VSV [42], attenuated VSV [43], Influenza transcription replication kinetics [44] and HCV replication kinetics [45]. Our goal was to find a set of mechanisms that would allow transcription while maintaining the polymerase association with the template as shown experimentally. We conclude that sliding on the N-RNA template would be essential for efficient transcription of NNS RNA viruses.

Understanding how polymerases initiate and sustain transcription is attractive both as a potential antiviral target and also because these polymerases represent an almost infinite dilution transcription machinery. Understanding the mechanics of this machine therefore may open doors to nano-technological applications.

## Materials and Methods

### Monte Carlo simulations

Negative stain electron microscopy of single VSV polymerases shows them as 10 nm ring-like structures which can adopt different conformations with appendages [7], [19]. At our low-resolution approximation, we divided the genome template into 10 nm segments and at any given time, polymerases could occupy any of these segments, this approximation resulted in n_Total_ = 300 segments along the template (n = 1 to 300). There are 50 polymerases that occupy the genome template L_i_ (i = 1 to 50). No two polymerases can occupy the same segment simultaneously. To create a Monte Carlo simulation, we also digitized the time domain. The essential time scale for the simulation τ is the time required for transcribing one segment of the N-RNA by a polymerase. Given an average rate of transcription of 3.7 nt/s [15], we calculate τ = 7.8 Sec. Based on this calculation, we set up our Monte Carlo simulation in which 50 polymerases (L_i_) were tracked along the digitized genome template with incremental time τ. In each time step all the polymerases are forced to choose a reaction with the following rules:

1) Transcription is initiated only if the polymerase is on the TIS; 2) Transcription follows a linear trajectory during each time step τ, with 30% fall off rate at the end of each gene junction; 3) Non-transcribing polymerases move along the genome randomly, with a step size calculated based on the mean square displacement associated with a 1D random walker with a diffusion coefficient D_sl_; 4) Collision of non-transcribing polymerases results in reflection of trajectories without dissociation from the template; 5) Reflective boundary conditions are set up at the ends of the template for non-transcribing polymerases; 6) Non-transcribing polymerases fall off the template with a rate K_off_ and can immediately rebind back with a rate K_hopp_ (S1 Text) or can stay in solution till next time step; 7) Free polymerase in solution would bind back to the template with a rate K_on_, once binding back, they have a higher probability of binding to the TIS compared to a regular template section with a factor R.

K_on_ is not determined experimentally, however an upper limit is calculated based on the diffusion-limited reaction. The upper limit of a diffusion limited reaction is 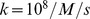. For a single polymerase and a single template in the volume of cytosol, the maximum reaction rate would be:
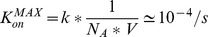



To incorporate the stochastic binding within the Monte Carlo simulations, we used a Gillespie Algorithm (SSA) [46] to determine a stochastic binding time t_binding_ based on the K_on_ in the simulation. 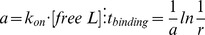
r is a pseudorandom number drawn from the uniform distribution on the open interval (0,1).

Each polymerase that falls off the template will be assigned its own t_binding_ time, and will be kept in solution until the simulation time exceeds t_binding_ time. The polymerase then gets reassigned to an available site on the template. The parameter R, which is the ratio of binding strength between TIS and unspecific sites, determines the probability of polymerase binding to the TIS P_TIS_ =  (R/(n_Total_+R)) or the probability of randomly binding on the template P_nonspecific_ =  (n_Total/_(n_Total_ +R)) in which n_Total_ is the total number of available binding sites.

In the described simulations with no sliding, we could not reconcile a healthy transcription rate, which produces ∼100 N mRNA in 1 hr [15] with the 20 pM dissociation constant of the polymerases from the template (S1 Text).

## Supporting Information

S1 Figure **Analysis of VSV proteins binding to RNPs.** VSV proteins were sequentially extracted in low then high salt buffers after virions lysis. Soluble and RNPs-bound factors were fractionated by ultra-centrifugation on 20% glycerol cushion, and analyzed by SDS-PAGE and proteins staining. VSV: full virions; 1–10: fractions collected from top to bottom of tubes after ultra-centrifugation, respectively; RNPs: pelleted RNPs, respectively.
(TIF)

S2 Figure **Western blots of varying dilutions of RNP and Fraction 1.** The following volumes of Fraction 1 were loaded: 1 = 30 µl; 2 = 20 µl; 3 = 10 µl; 4 = 5 µl; 5 = 2.5 µl; 6 = 1 µl. RNPs: 1 µl from each dilution 1 = 1/1; 2 = 1/5; 3 = 1/10; 4 = 1/50; 5 = 1/100.
(TIF)

S3 Figure **Linear genome (asymmetric versus randomly distributed initial conditions).** Number of N mRNA after one hour of transcription versus various dissociation rates and different 1-D diffusion coefficients under different promoter strength R: B). R = 1 C). R = 500. The morphology of N-RNA template is linear and initially all 50 L are located either asymmetrically at the 5′ end of the genome (dark grey) or randomly along the template (light grey). Under R = 500, D_sl_ = 10^4^ nm^2^/s and K_off_ = 10^−5^/s, random distribution produced 86.2±3.7 N mRNA compared to production of 72.8±7.7 with asymmetric distribution.
(TIF)

S4 Figure **Monte Carlo simulations of UV irradiated genome templates.** Monte Carlo simulations of 50 polymerases on a linear genome template with a damage site on the end of N gene, (A) shows a representation of this model with collision rules resulting in release of non-transcribing polymerases after collisions, and also template damage rules: the transcribing polymerase is either stuck (I) or released (II) at the damage site. (B) The calculated N mRNA amounts within one hour of simulations for various sliding D_sl_ and dissociation K_off_ rates with relative TIS binding affinity R = 500. (C) Number of Polymerase that remain on the template vs Time (R = 500, D_sl_ = 10^4^ nm^2^/s and K_off_ = 10^−5^/s). Green line follows template damage rule I, and grey line follows rule II.
(TIF)

S1 Text **Supplemental information.** This file includes dissociation constant measurements and details of the Monte Carlo Simulations.
(DOCX)
