# Supplementary figures and images for: Vesicular Stomatitis Virus Polymerase's Strong Affinity to Its Template Suggests Exotic Transcription Models
Source: PLoS Comput Biol. 2014 Dec 11;10(12):e1004004. doi: 10.1371/journal.pcbi.1004004 (PMC4263359; doi:10.1371/journal.pcbi.1004004)

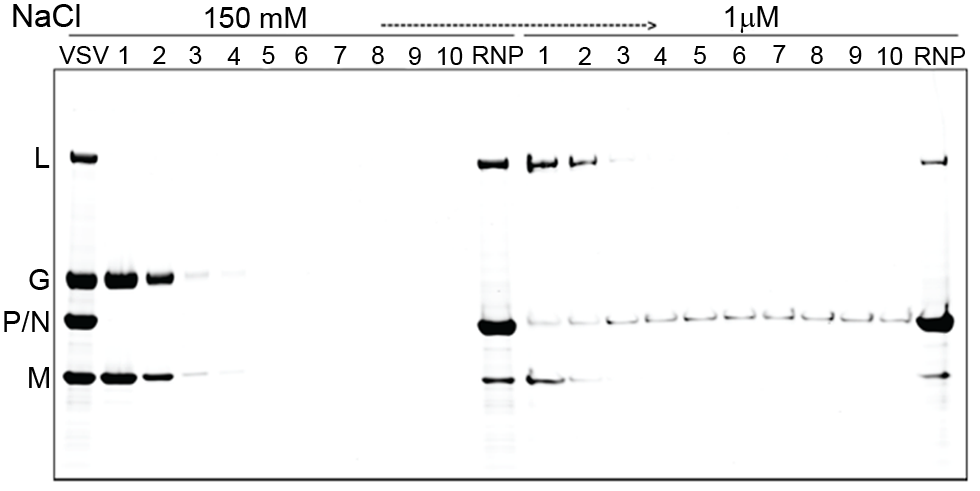

Supplement: S1 Figure — Analysis of VSV proteins binding to RNPs. VSV proteins were sequentially extracted in low then high salt buffers after virions lysis. Soluble and RNPs-bound factors were fractionated by ultra-centrifugation on 20% glycerol cushion, and analyzed by SDS-PAGE and proteins staining. VSV: full virions; 1–10: fractions collected from top to bottom of tubes after ultra-centrifugation, respectively; RNPs: pelleted RNPs, respectively. (TIF) [file pcbi.1004004.s001.tif]

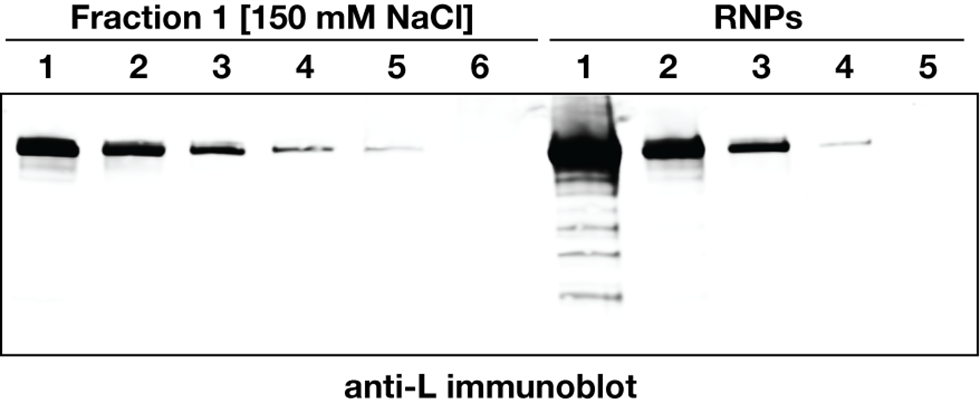

Supplement: S2 Figure — Western blots of varying dilutions of RNP and Fraction 1. The following volumes of Fraction 1 were loaded: 1 = 30 µl; 2 = 20 µl; 3 = 10 µl; 4 = 5 µl; 5 = 2.5 µl; 6 = 1 µl. RNPs: 1 µl from each dilution 1 = 1/1; 2 = 1/5; 3 = 1/10; 4 = 1/50; 5 = 1/100. (TIF) [file pcbi.1004004.s002.tif]

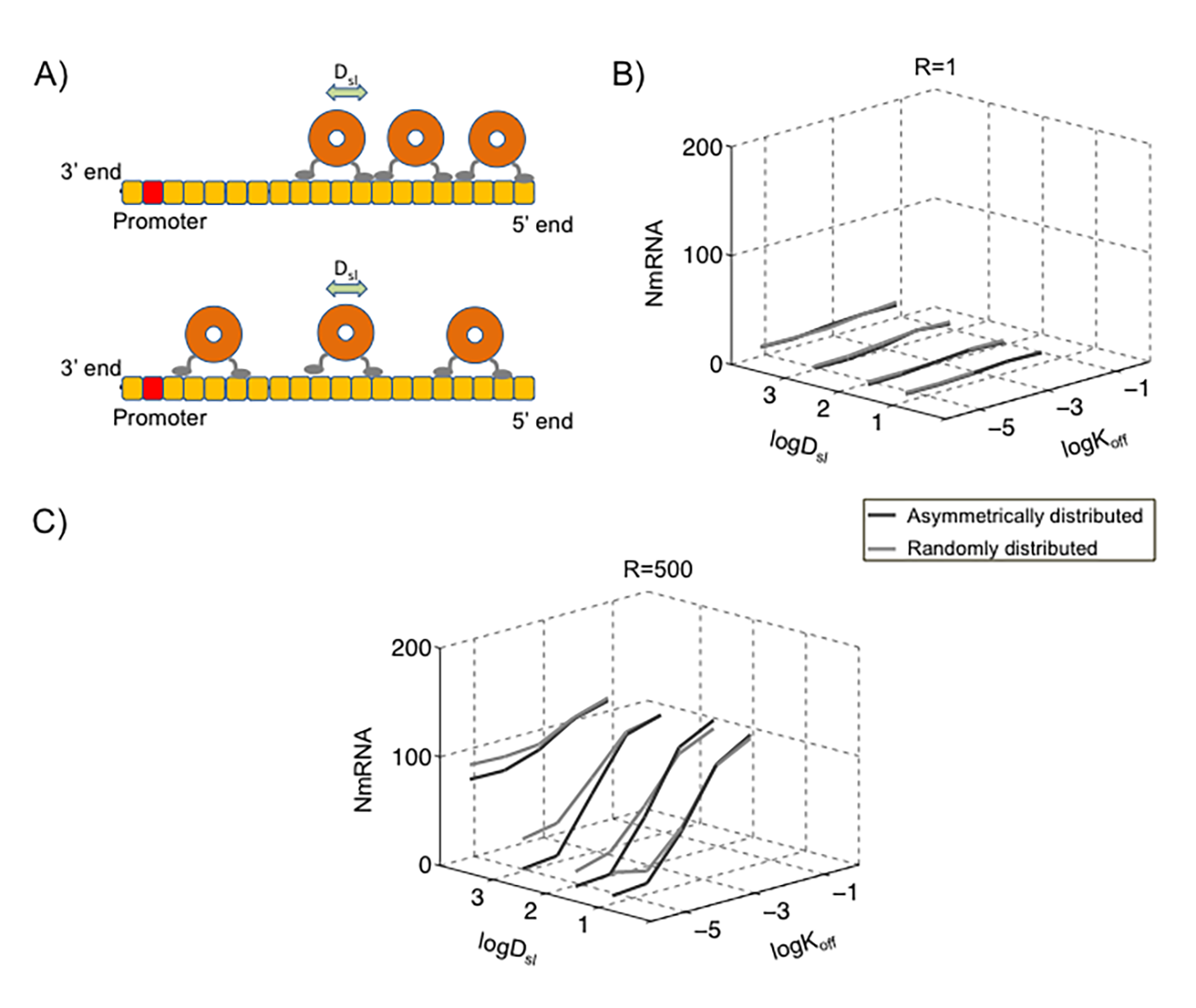

Supplement: S3 Figure — Linear genome (asymmetric versus randomly distributed initial conditions). Number of N mRNA after one hour of transcription versus various dissociation rates and different 1-D diffusion coefficients under different promoter strength R: B). R = 1 C). R = 500. The morphology of N-RNA template is linear and initially all 50 L are located either asymmetrically at the 5′ end of the genome (dark grey) or randomly along the template (light grey). Under R = 500, Dsl = 104 nm2/s and Koff = 10−5/s, random distribution produced 86.2±3.7 N mRNA compared to production of 72.8±7.7 with asymmetric distribution. (TIF) [file pcbi.1004004.s003.tif]

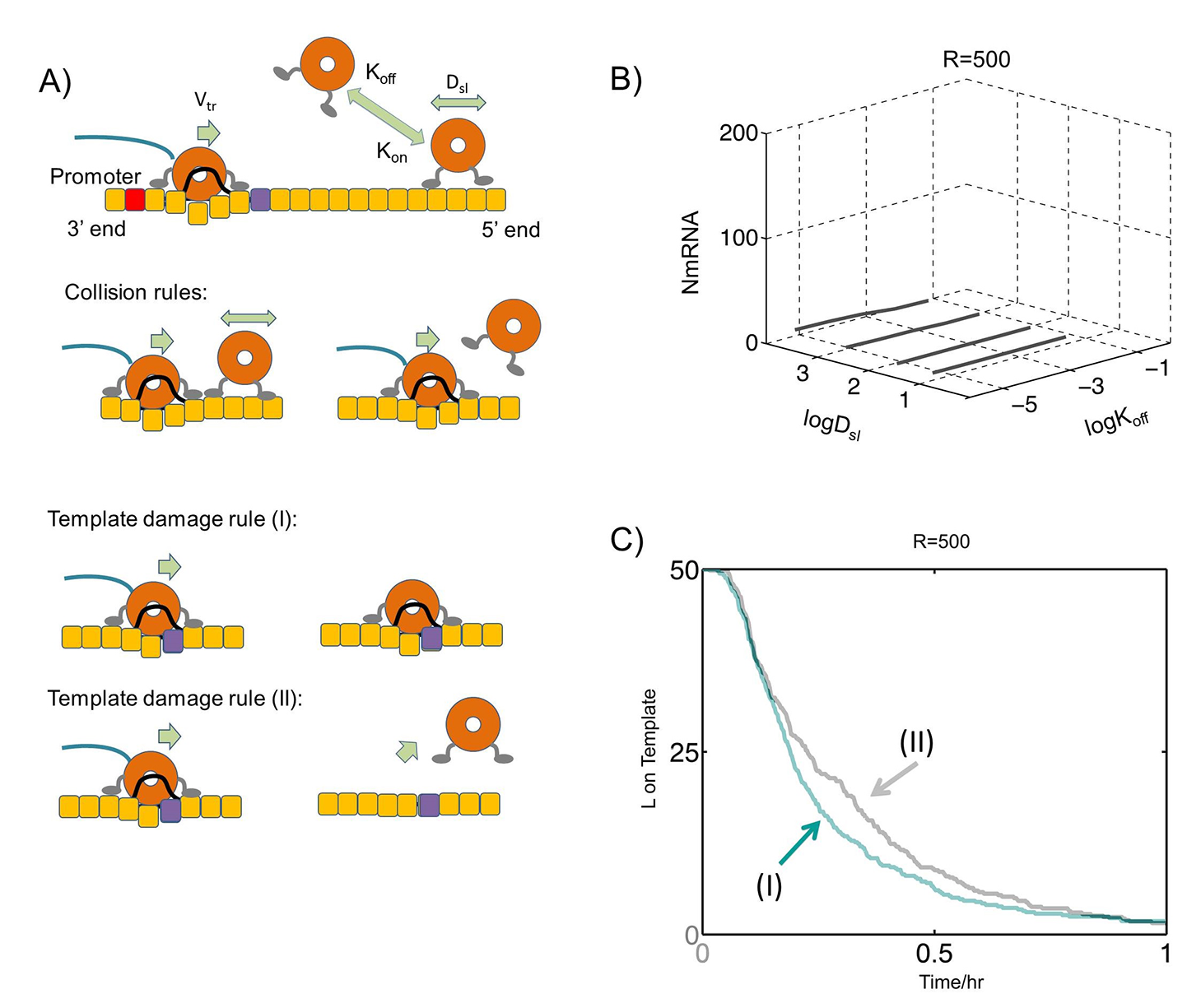

Supplement: S4 Figure — Monte Carlo simulations of UV irradiated genome templates. Monte Carlo simulations of 50 polymerases on a linear genome template with a damage site on the end of N gene, (A) shows a representation of this model with collision rules resulting in release of non-transcribing polymerases after collisions, and also template damage rules: the transcribing polymerase is either stuck (I) or released (II) at the damage site. (B) The calculated N mRNA amounts within one hour of simulations for various sliding Dsl and dissociation Koff rates with relative TIS binding affinity R = 500. (C) Number of Polymerase that remain on the template vs Time (R = 500, Dsl = 104 nm2/s and Koff = 10−5/s). Green line follows template damage rule I, and grey line follows rule II. (TIF) [file pcbi.1004004.s004.tif]
