# Supplementary material for: Vesicular Stomatitis Virus Polymerase's Strong Affinity to Its Template Suggests Exotic Transcription Models
Source: PLoS Comput Biol. 2014 Dec 11;10(12):e1004004. doi: 10.1371/journal.pcbi.1004004 (PMC4263359; doi:10.1371/journal.pcbi.1004004)
Supplement: S1 Text — Supplemental information. This file includes dissociation constant measurements and details of the Monte Carlo Simulations. (DOCX) [file pcbi.1004004.s005.docx]

**Supplemental Material:**

**Calculating the dissociation constant of the polymerase (L) from the genome template (RNP)**

VSV wild type plaques were amplified in 175 cm^2^ monolayers of BHK-21 cells 24 hours at 37C. Viral supernatants were pre-cleared by centrifugation 10 min at 3,000 rpm, filtrated through 0.45 µm, then VSV particles were pelleted 1.5 hours at 43,000 xg in a Beckman SW41 rotor. VSV pellets were re-suspended in PBS and ultra-centrifuged at 111,000 xg for 3 hours on 5-60% sucrose gradient. VSV layers were extracted and pelleted on 10% sucrose cushion at 111,000 xg for 1 hour. VSV pellets (measured 2 mg) were re-suspended in lysis buffer (20 mM Tris-HCl [pH 8], 150 mM NaCl, 1.8% Triton X-100, 1 mM 1,4-dithiothreitol) and incubated 12 hours at 4C. The total volume of the lysis was 1.5ml. Lysed virions were loaded on 20% glycerol cushion and ultra-centrifuged at 111,000 xg for 3 hours to separate free soluble proteins from RNP complexes. RNPs were further extracted with 1 M NaCl 12 hours at 4C, reloaded on the 20% glycerol cushion, and ultra-centrifuged at 111,000 xg for 3 hours. Fractions (each 1ml in volume) were collected from top to bottom of tubes, and analyzed by SDS-PAGE and proteins staining as shown in Figure S1.

As shown in Figure S1, under 150 mM salt, VSV proteins M and G became solubilized during the lysis and therefore collected at the first fraction of the 20% Glycerol spin. The majority of the L protein however travels along with the RNP to the bottom of the 20% glycerol and is visible along with the RNP fraction. The free L protein should be in Fraction 1, however the protein gel does not have the sensitivity for its detection. To detect the level of free L, we loaded varying amounts of RNPs as well as Fraction 1 extracts on a separate protein gel and performed western blots as shown in Figure S2. Using the western blots at higher volumes, we can detect the Free L protein within the fraction 1. Based on the densitometry of the western blots, the amount of free L in fraction 1 is 1/100 the amount of L associated with the RNPs. Based on this data we calculated the dissociation constant as follows:

Based on the protein and lipid composition of the VSV we calculated that 2% of virion mass is the L protein. Therefore the total mass of L protein within the lysis was 2 mg*0.02. Based the volume of the lysis buffer (1.5ml ) and the mass of L, we calculated the concentration of total L in the lysis buffer as 100 nM. Based on the above densitometry measurements, the concentration of free L was therefore 100 nM * 0.01 =1 nM.

In average each VSV virion packages 50 L proteins along with one genome template (RNP). Therefore the concentration of the template was calculated as 100 nM /50= 2 nM. Based on these concentration the dissociation constant was calculated as:

$$K_{d}=\frac{\left[ L \right]\left[ RNP \right]}{\left[ L+RNP \right]}=\frac{1nM 2nM}{100 nM}=20 pM$$

**Calculating the limits of K_hopp_**

For calculating K_hopp_, only very short time scales (< µSec) are considered. A polymerase can bind back to the template due to immediate collision back with the template defined by K_hopp_. K_hopp_ is calculated as follows: Taken the site where the polymerase falls off as the origin $S_{1}$, then the probability of this polymerase to find another site $S_{2}$on the template from site $S_{1}$ through 3-D diffusion at time t is calculated precisely by:

$P\left( S_{2},t | S_{1,},0 \right)=\iiint^{\infty} \sqrt[3]{\frac{3}{2\pi Na^{2}}}e^{-\frac{3r^{2}}{2Na^{2}}}*\frac{1}{\gamma}e^{-\frac{r^{2}}{4Dt}}*\frac{S}{4\pi r^{2}}dr$ (1)

Eq.1 is composed of three parts: first the probability that the two sites are separated by a distance r considering the N-RNA template as an ideal worm like chain model, a is the Kuhn length and is approximated as twice of the persistence length estimated from electron microscopy of purified templates (a = 3 nm)[1]. Second term in (1) describes the possibility of a polymerase diffusing a distance r after time t through 3D diffusion, γ is a normalization constant, D is 3D diffusion coefficient, $(D=3.4\times{10}^{7}{nm}^{2}/s)[Lawrence, 1994 \#36]$; Third, the probability of finding the specific site on the whole space. Based on Eq. 1, the probability of finding a separate section of the template far from the detachment site is almost zero; the probability of finding the template has a FHMW of 40nm. The actual sum of the probability shown in Eq3 on the whole template becomes 0.07. To simplify incorporation of hopping in our model, we assumed that when the polymerase falls off the template, it will have 7% chance of rebinding back to the template within 4 adjacent binding sites which defines the K_hopp_.

[1] A. Desfosses, E. A. Ribeiro, G. Schoehn, D. Blondel, D. Guilligay, M. Jamin, R. W. H. Ruigrok, and I. Gutsche, Nat Commun **4**, 1429 (2013).
